# Supplementary material for: A set of multi-entry identification keys to African frugivorous flies (Diptera, Tephritidae)
Source: Zookeys. 2014 Jul 24;(428):97–108. doi: 10.3897/zookeys.428.7366 (PMC4143993; doi:10.3897/zookeys.428.7366)
Supplement: Supplementary material 6 — Key to Ceratitis [file zookeys-428-097-s006.zip › SF6_ZooKeys_key to Ceratitis/key/SF6_ZooKeys_key to Ceratitis/Media/Html/Ceratitis rubivora .htm]

Ceratitis (Pterandrus) rubivora Coquillett


***Ceratitis*** **(*Pterandrus*) *rubivora*
Coquillett**

*Ceratitis rubivora* Coquillett, 1901:
29.

Body
length: 4.56 (3.95-5.00) mm; wing length: 4.98 (4.45-5.45) mm.

Male

Head: Antenna yellow. First flagellomere 2-3 times as long
as pedicel. Arista with short to moderately long rays; ventral rays shorter and
sparser than dorsal rays, especially basally. Frons pale, in center more
yellow; with short scattered setulae of same color as frons. Frontal setae well
developed, occasionally anterior one slightly underdeveloped. Face white. Genal
seta and setulae dark or dark reddish, latter moderately to poorly developed.

Thorax:
Postpronotal lobe yellowish white, without spot, at most with darker yellow
coloration near postpronotal seta. Scutal pattern: ground color, grayish-brown,
usually with orange tinge, sometimes without; with streaks and darker markings
but without distinct spots or clearly defined stripes, except prescutellar
white separate markings, with paler gray area in between. Scapular setae dark
reddish or black. Scutellum yellowish white, basally with ill defined separate
dark spots (occasionally well developed), apically with three separate black
spots, extending to basal 0.33, sometimes only to half-way. Anepisternum on
ventral half yellowish orange to brown; setulae pale.

Legs:
Yellow except where otherwise noted; setation typical for subgenus, mixed pale
and dark. Foreleg: femur anteriorly with dorsal margin brownish over entire
length; posterodorsally brown, with dispersed long dark setulae along entire
length, but not forming distinct bush; ventral setae pale, distally dark,
rarely dark over entire length. Midleg: femur brown on distal half to 0.66
(usually only ventral part brown anteriorly) except at apical margin where
white, with weak silvery shine; ventrally with dark feathering along distal
half, basally with more dispersed pale or dark setulae; tibia broadened,
largely brownish black except at extremities, with conspicuous silvery shine
when viewed from certain angle, with black feathering dorsally along distal
0.8-0.9 and ventrally along distal 0.8. Hindleg: femur partly brownish colored;
at apical 0.25 with longer setulae dorsally and ventrally. Darker
coloration of legs in some specimens more pronounced and more extensively
blackish.

Wing:
banding yellowish brown. Interruption between marginal and discal bands
near vein R_1_ clear and complete; discal band often partly,
occasionally fully interrupted in discal cell; cubital band free; medial band
free (not always well defined, sometimes missing); crossvein R-M at or just
proximal to middle of discal cell. Apex of vein R_1_ distal to level
of crossvein R-M. Crossvein DM-Cu oblique anterobasally.

Abdomen:
Mostly yellow. Tergite 1 with black patches across posterior margin. Tergites 2
and 4 with pale gray band on posterior half; tergite 4 with anterior margin
brown. Tergite 3 with brown transverse band along� posterior half to 0.66;
tergite 5 with brown band along anterior 0.33 and narrowly brownish black
across posterior margin. Brown bands on tergites 3, 4 and 5 often interrupted
in middle. Male epandrium in lateral view with posterior lobe of lateral
surstylus short and slightly curved, anterior lobe pronounced.

Female

As
male except for the following characters: First flagellomere more yellowish
orange. Gena sometimes darker yellow. Genal setula and seta black and well
developed. Scapular seta black. Anepisternal pilosity rarely with few dark
setulae. Legs without feathering; femora yellow, often with dark patches;
forefemur posteroventrally with pale pilosity. Discal band complete. Oviscape
shorter than preabdomen. Aculeus at most five times longer than wide; tip
pointed and lateral margin straight.

(Description
after De Meyer & Freidberg, 2006)
